# Concurrent gene alterations with EGFR mutation and treatment efficacy of EGFR-TKIs in Chinese patients with non-small cell lung cancer

**DOI:** 10.18632/oncotarget.15337

**Published:** 2017-02-15

**Authors:** Wentao Hu, Yahui Liu, Jian Chen

**Affiliations:** ^1^ Department of Thoracic Surgery, Ningbo First Hospital, Ningbo, Zhejiang 315000, P.R. China; ^2^ Key Laboratory of Ningbo, Ningbo First Hospital, Ningbo, Zhejiang 315000, P.R. China

**Keywords:** epidermal growth factor receptor, tyrosine kinase inhibitor, non-small cell lung cancer, concurrent gene, efficacy

## Abstract

**Purpose:**

We investigated the frequency of concurrent genes in *EGFR*-mutant non-small cell lung cancer patients and determined its value in predicting the efficacy of EGFR-TKIs treatment.

**Methods:**

Three hundred and twenty patients, who harbored *EGFR* activating mutations and received EGFR-TKIs treatment, were examined for another eight genes including *KRAS*, *NRAS*, *PIK3CA*, *BRAF*, and *HER2* mutations and *ALK*, *ROS1*, and *RET* fusion genes based on reverse transcription PCR. Progression-free survival and overall survival with EGFR-TKIs treatment were evaluated using Kaplan-Meier methods and compared between different patients using log-rank tests.

**RESULTS:**

Twenty-one (6.6%) of 320 *EGFR* mutant samples with additional gene alterations were identified. The most common concurrent gene was *PIK3CA* mutation (n = 9), followed by *EML4*-*ALK* rearrangement (n = 6), *HER2* mutation (n = 3), *RET* rearrangement (n = 1), *ROS1* rearrangement (n = 1) and *KRAS* mutation (n = 1). Patients with single *EGFR* mutation had a significantly longer progression-free survival than those with concurrent genes (10.9 vs. 6.0 months, *P* = 0.002). Among the 21 cases, patients with *PIK3CA* mutation had the longest median progression-free survival (7.6 months), followed by *ALK* rearrangement (5.0 months) and other gene types (1.2 months). No overall survival difference was found between patients with single EGFR mutation and concurrent gene alterations (21.0 vs.17.6 months, *P* = 0.17).

**Conclusion:**

We demonstrated that concurrent gene alterations occurred in some patients with *EGFR* mutations. Concurrent gene alterations decreased the efficacy of EGFR-TKIs.

## INTRODUCTION

Epidermal growth factor receptor (*EGFR*) mutations occur in about 40% to 50% of lung adenocarcinoma patients of East Asian descent [1, 2]. The median progression-free survival (PFS) is approximately 9 to 13 months and the objective response rate of 60% to 70% in patients carrying *EGFR* mutations treated with EGFR-TKIs [3–6].

Drug resistance is a big issue for most patients with clinically evident non-small cell lung cancer (NSCLC). T790M mutation, *MET* amplification and *PIK3CA* mutations contributed to secondary resistance to EGFR-TKIs and several new drugs targeting resistance have emerged [7–12]. Primary resistance is another challenge in clinical practice, however, the mechanism is not well investigated currently. Coexistent genetic alterations in cancer-driving genes, i.e., *KRAS* mutations, *PTEN* loss and *BIM* polymorphisms were identified to be associated with primary resistance for EGFR-TKIs treatment [13–14]. But, most studies focused on concurrent *ALK* and *EGFR* mutations [15–16]. Other genes such as *PIK3CA* and *HER2* were not well reported. The efficacy of EGFR-TKIs for NSCLC patients with coexisting genetic alterations remains unclear.

In the present study, we used multiple gene screening of 320 NSCLC patients harboring EGFR-sensitive mutations and evaluated the frequency of concomitant genetic alterations, further to investigate the efficacy of EGFR-TKIs treatment in these patients.

## RESULTS

### Patient characteristics

The characteristics of the 320 patients are shown in Table 1. Twenty-one patients with EGFR mutation that harbored a concurrent driver gene were identified. The clinical and molecular characteristics of the 21 patients are shown in Table 2. There were 11 male and 10 female with a median age of 62 years. Twenty patients presented with a histology of adenocarcinoma and one typical of adenosquamous carcinoma. Seven patients included former or current smokers and 14 were never-smokers. No clinical or pathological differences were observed between patients with single *EGFR* mutation and those who harbored concurrent genes (Table 3).

**Table 1:** Demographic characteristics of the study population (n=320)

	Number
Gender	
Male	176
Female	144
Age	
Range	31-78
Median	59
<60	196
≥60	124
Smoking status	
Never	197
Former/current	123
Histology	
Adenocarcinoma	302
No-adenocarcinoma	18
Stage at EGFR-TKI treatment	
IIIB	5
IV	315
Surgical history	
Yes	135
No	185
Type of EGFR-activating mutation	
Exon 19 deletion	157
Exon 21 L858R	142
Exon 18 G719X	8
Exon 21 L861Q	5
Other mutation	8
Concurrent mutation	
Yes	21
No	299
EGFR-TKIs	
Erlotinib	43
Gefitinib	56
Icotinib	220
Afatinib	1
EGFR-TKIs in which line	
First-line	76
Second-line	189
Third-line or further-line	55
Performance score at EGFR-TKI treatment	
0-1	249
2-3	71

**Table 2:** Clinical profile of concurrent gene alterations in non-small cell lung cancer patients

Case	Gender	Age	Smoking history	Histology	Gene type	EGFR-TKI/which line	Response	PFS/month	OS/month
1	Male	44	Yes	Adenocarcinoma	19del+PIK3CA	Gefitinib/Second	PR	10.4	18.7
2	Male	75	No	Adenocarcinoma	19del+PIK3CA	Icotinib/Third	PR	11.2	20.3
3	Female	62	No	Adenocarcinoma	L861Q+PIK3CA	Icotinib/Second	PD	1.2	12.5
4	Male	59	Yes	Adenocarcinoma	19del+PIK3CA	Icotinib/Third	PR	7.6	17.6
5	Female	75	No	Adenocarcinoma	19del+PIK3CA	Gefitinib/Third	PR	6	15.5
6	Male	62	No	Adenocarcinoma	L858R+PIK3CA	Icotinib/Second	SD	9.5	16.7
7	Male	67	Yes	Adenosquamous	19del+PIK3CA	Icotinib/Third	PR	9.7	20.5
8	Female	66	No	Adenocarcinoma	G719X+PIK3CA	Icotinib/Second	PD	2	12.1
9	Male	44	Yes	Adenocarcinoma	19del+PIK3CA	Gefitinib/Second	PR	7.5	17.6
10	Female	64	No	Adenocarcinoma	L858R+ALK	Gefitinib/First	SD	4.5	16.5
11	Female	40	No	Adenocarcinoma	19del+ALK	Icotinib/Second	PR	8.9	24.3+
12	Male	64	No	Adenocarcinoma	19del+ALK	Erlotinib/First	PR	14	28.7
13	Female	59	No	Adenocarcinoma	19del+ALK	Icotinib/Third	PD	1.2	19.5
14	Male	45	No	Adenocarcinoma	19del+ALK	Erlotinib/First	SD	5	18.6
15	Male	64	Yes	Adenocarcinoma	19del+ALK	Icotinib/Second	SD	6.5	17.7
16	Female	65	No	Adenocarcinoma	19del++HER2	Icotinib/Third	PD	1.2	12.5
17	Female	69	No	Adenocarcinoma	L861Q+HER2	Icotinib/Second	PD	1	6.5
18	Female	50	No	Adenocarcinoma	19del+HER2	Icotinib/Second	PR	14.4	17.7
19	Female	60	No	Adenocarcinoma	L858R+RET	Gefitinib/Third	PD	2.2	10.2
20	Male	63	Yes	Adenocarcinoma	L858R+KRAS	Erlotinib/First	PD	1	6.5
21	Male	49	Yes	Adenocarcinoma	19del+ROS1	Erlotinib/First	PR	24	47.8+

**Table 3:** Comparative analysis of clinical profile between single EGFR mutation and concurrent gene alteration patients

Characteristics	Single EGFR mutation	Concurrent alteration	*P*
Gender			0.80
Male	165	11	
Female	134	10	
Age			0.02
<60	188	8	
≥60	111	13	
Smoking status			0.62
Never	183	14	
Former/current	116	7	
Histology			0.73
Adenocarcinoma	288	20	
No-adenocarcinoma	11	1	
Stage at EGFR-TKI treatment			0.75
IIIB	5	0	
IV	294	21	
EGFR mutation type			0.57
Exon 19 deletion+Exon 21 L858R	280	19	
Other types	19	2	
Performance score at EGFR-TKI treatment			0.20
0-1	235	14	
2-3	64	7	

### Gene results

Among the 320 patients with EGFR mutations, 157 were with deletion in exon 19, 142 with L858R point mutation in exon 21,13 with L861Q or G719X mutation in exon 18, and 8 with other mutations (three of T790M mutation, four of 20 insertion and one of S768I). All the 320 patients were analyzed for *KRAS, NRAS, PIK3CA, BRAF, HER2* mutations and *ALK, ROS1*, and *RET* fusion genes. Coexisting mutations or fusions were identified in 21 patients (6.6%). This analysis included *PIK3CA* mutation (n = 9, 42.9%), followed by *EML4-ALK* rearrangement (n = 6, 28.6%), *HER2* mutation (n = 3, 14.3%), *KRAS* mutation (n = 1, 4.8%), *RET* rearrangement (n = 1,4.8%), *ROS1* rearrangement (n = 1,4.8%), *BRAF* mutation (n = 0, 0%), and *NRAS* mutation (n = 0, 0%). The coexisting mutations are listed in Table 2. Among the 21 patients, 14 included those with deletion in exon 19, 4 with L858R mutation in exon 21, one with G719X mutation in exon 18 and one with L861Q mutation in exons 21. More frequency of coexisting mutations in deletion in exon 19 was observed than L858R mutation in exon 21 (8.9% vs.2.8%, *P*=0.028).

### Efficacy analysis

One hundred and eighty-six patients with single *EGFR* mutation showed partial responses (62.2%), one with complete response (0.3%) and 67 showed stable disease (22.4%); 46 patients had progressive disease. The ORR was 62.5% and DCR was 84.9%, In patients with concurrent gene alterations, the ORR and DCR were 47.6% and 66.7%, respectively. The efficacy comparisons are shown in Table 4.

**Table 4:** Clinical efficacy comparison of EGFR-TKI in single EGFR mutation and concurrent gene alterations

Best response	Single EGFR mutation (n=299)	Concurrent gene alterations (n=21)	*P*
CR	1(0.3%)	0(0.0%)	0.79
PR	186(62.2%)	10(47.6%)	0.18
SD	67(22.4%)	4(19.0%)	0.25
PD	46(15.4%)	7(33.3%)	0.03
ORR	62.5%	47.6%	0.17
DCR	84.9%	66.7%	0.03
Median PFS(month)	10.9	6.0	0.002
Median OS(month)	21.0	17.6	0.17

**Figure 1:**
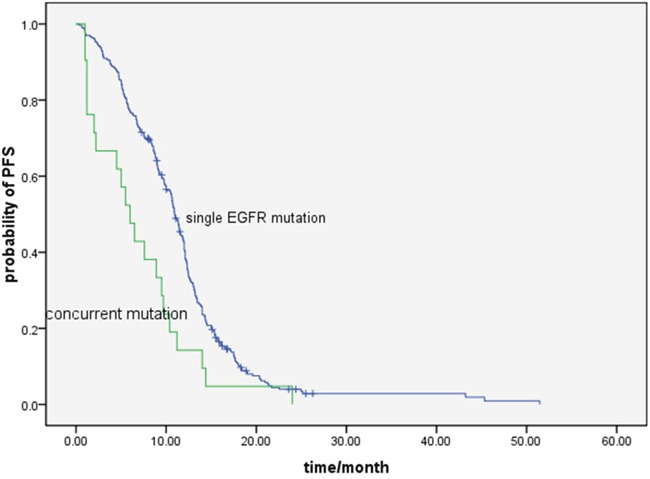
Comparison of progression free survival with EGFR-TKI treatment between single EGFR mutation and concurrent gene alterations patients (10.9 vs.6.0 months, *P*= 0.002)

The median PFS in all the 320 patients was 10.8 months (95%CI, 9.9-11.6). The PFS in the group with single *EGFR* mutation and concurrent gene alterations group were 10.9 months (95%CI,10.0-11.5) and 6.0 months (95%CI,3.8-8.2), respectively (*P* = 0.002) (Figure 1). The median PFS in patients carrying *PIK3CA, ALK* and other genes were 7.6 months, 5.0 months and 1.2 months, respectively (*P*=0.880). No PFS difference was found between *EGFR/PIK3CA* mutation and *EGFR*/other gene concurrent patients (*P*=0.881).

The PFS in the group with single *EGFR* exon 19 deletion mutation and concurrent gene alterations group were 11.4 months (95%CI,10.4-12.5) and 6.0 months (95%CI, 4.1-7.8) (*P* = 0.001). The PFS in the group with single *EGFR* exon 21 L858R mutation and concurrent gene alterations group were 9.5 months (95%CI, 8.3-10.8) and 2.2 months (95%CI, 0.0-5.9) (*P* = 0.009).

A multivariate Cox regression model was constructed with the incorporation of age, gender, performance status, and mutation types (single vs.concurrence) to evaluate the PFS. Mutation types (*P*=0.032) remained as independent factor for PFS.

**Figure 2:**
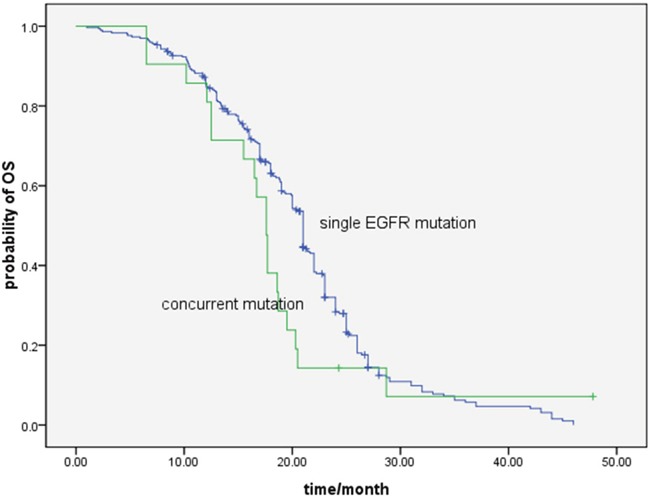
Comparison of overall survival with EGFR-TKI treatment between single EGFR mutation and concurrent gene alterations patients (21.0 months vs.17.6 months, *P*=0.170)

The median survival time of all the patients was 21.0 months (95%CI,19.5-25.4). The OS in the single EGFR mutation and concurrent gene alterations group was 21.0 months, and 17.6 months, respectively (*P* = 0.170) (Figure 2).

## DISCUSSION

Our data demonstrated that the frequency of co-alterations between EGFR and other driver genes (ALK, ROS1, RET, PIK3CA, BRAF, KRAS, NRAS, BRAF) in NSCLC was 6.6%. Patients of NSCLC without concurrent gene had a significantly longer PFS with EGFR-TKIs treatment. To the best of our knowledge, this is the first study demonstrating the presence of EGFR mutations concurrent with multple gene mutations and the therapeutic efficacy of EGFR-TKIs.

Although driver genes in NSCLC were reported to be mutually exclusive [18–20], several studies have shown that driver genes occur concurrently with EGFR mutations [21–22]. In the current cohort, the frequency of concurrent EGFR/ALK mutations is 1.9%, which is consistent with previous studies reporting in the range of 0.0% to 6% [23–24]. The phosphatidylinositol 3-kinase (PI3K) plays an important role in cancer cell metabolism and proliferation. PIK3CA mutations are commonly found in a variety of cancers, with a prevalence of about 2% to 4% in NSCLC [22–25]. PIK3CA mutations co-exist mostly with KRAS mutations in lung cancer. However, the underlying mechanisms involving EGFR mutation are unclear [22]. A report by Chaft et al. included 23 lung adenocarcinoma patients with PIK3CA and 3 with EGFR concurrent mutations [26]. In the present study, 2.8% of NSCLC patients in China with EGFR mutations harbored PIK3CA mutations. With the emerging of next generation sequencing(NGS), more and more concurrent genes were observed. One study reported by Kim et al. showed that compound EGFR mutation was frequently detected with co-mutations of EGFR actionable genes by NGS [27].

The frequency of concomitant *EGFR* mutations and other driver genes except *ALK* and *PIK3CA* in NSCLC were not well known, and elucidated the case report [28–29]. In our cohort, three patients with *HER2*, one with *RET*, one with *KRAS* and one with *ROS1* gene were found, while, no *BRAF* and *NRAS* were found coexisting with *EGFR* mutation.

The efficacy of EGFR-TKIs treatment in patients with concomitant *EGFR* mutations and other driver genes is not well studied due to their rarity. The median PFS of EGFR-TKIs was 11.2 months in Yang’s study including 10 patients with co-existing *EGFR*/*ALK* mutations [30]. Relative levels of phospho-EGFR predicted the efficacy of EGFR-TKI in patients with *EGFR*/*ALK* mutations. In the current series, the median PFS of six patients with concurrent *EGFR*/*ALK* mutations was 5.0 months, which is shorter than in Yang’s study. The small sample may explain the difference of our cases and previous studies. A concurrent *PIK3CA* mutation did not decreased the efficacy of EGFR-TKIs in Eng, et al. study [31], which including 10 patients of *EGFR/PIK3CA* co-altered. In contrast, in present cohort, we found that patients’ concurrent *PIK3CA* mutation may decrease the PFS and objective response, consistent with previous preclinical studies [32]. Different from Eng, et al. study, our results indicated no significantly OS difference between patients with single EGFR mutation and concurrent genes. One reason may contribute to the small sample of patients with concurrent genes. Another reason may due to the influence of additional treatment after failure of EGFR-TKI therapy. In our cohort, four of six patients with *ALK* rearrangement received the crizotinib treatment after failure of EGFR-TKI treatment and three with partial response. However, no further treatment data were provided in Eng, et al study. The influence of concurrent genes to overall survival should be validated with large number patients in the future studies.

The most remarkable shortcomings of our study were related to the small sample size of concurrent genes. Secondly, *MET* amplification, mutation and other genes like *NTRK1* and *PTEN*, which may coexist with *EGFR* gene in EGFR-TKIs treatment-naive samples, were not detected in current study for lack of sufficient tumor tissues. Thirdly, only five cases were treated with inhibitor focus on the coexisting gene (four with ALK and one with ROS1), so, the clinical efficacy of further treatment after failure of EGFR-TKIs could not be fully evaluated. Lastly, age and performance score imbalance were found between the single *EGFR* mutation and coexisting gene group, which may influence the outcome analysis in present study (Table 3). However, as the first study investigating the role of multiple genes in *EGFR* mutant patients, the findings are clinically meaningful.

In conclusion, this is the first study to focus on the predictive value of concurrent *EGFR* and other mutations in driver genes for EGFR-TKIs treatment. It suggests that some of the genes concomitant with *EGFR* mutation might decrease the efficacy of EGFR-TKIs treatment. In the future, prospective studies must validate the efficacy of different therapies for concurrent gene alterations NSCLC patients.

## MATERIALS AND METHODS

### Patient selection

Four hundred and twenty-nine consecutive patients who carried sensitive *EGFR* mutations and underwent EGFR-TKIs treatment for advanced NSCLC at Ningbo First Hospital were screened between 2009 and 2013. Of the 429 patients, 109 were ineligible because of a lack of tumor tissue for analysis of 8 genes. In 320 patients with identified genes, 135 patients had formalin-fixed paraffin-embedded (FFPE) tumor tissue blocks obtained at the time of surgical resection, 157 were tissue biopsies, and 38 with malignant effusion. Among the 320 samples, 270 of the samples used for 8 genes detection were obtained from the remaining tissues of EGFR gene analysis and 50 were re-obtained before EGFR-TKIs treatment. Ethics Committee of Ningbo First Hospital approved this study and a written informed consent was obtained from each participant.

### Gene detection

Amplification refractory mutation system (ARMS)-based *EGFR* mutation detection kit (Amoy, Xiamen, China) was used to detect *EGFR* mutation in all patients. The ARMS kit is able to detect 29 mutations: three in exon 18 (G719A, G719C and G719S; the kit was unable to distinguish between these subtypes, which are referred to as G719X hereafter), 19 deletions in exon 19, two mutations in exon 20 (T790M, S768I), three insertions in exon 20, and two mutations in exon 21 (L858R, L861Q).

A microscopy was used to patients with EGFR-mutated ensure the tumor tissues analyzed had more than 20% tumor contents. Genomic DNA or RNA was extracted from tumor tissues according to standard protocols (RNeasy Mini Kit, and QiAamp DNA Mini Kit, Qiagen, Hilden, Germany). Briefly, the isolated RNA samples were used for reverse transcription into cDNA using Revert Aid First Strand cDNA Synthesis Kit (Fermentas, St Leon-Rot, Germany). Either genomic DNA or cDNA was used for PCR amplification and sequencing. *HER2, KRAS, NRAS, BRAF*, and *PIK3CA* were PCR amplified using genomic DNA. Cycle sequencing of the purified PCR products was carried out with PCR primers using the commercially available ADx Mutation Detection Kits (Amoy, Xiamen, China).

The *ALK*, *ROS1*, and *RET* fusion mRNAs were detected by PCR with fusion gene detection kit (Amoy, Xiamen, China). In brief, total RNA was extracted with QiagenRNeasy FFPE Kit. The mRNA was reverse-transcribed to cDNA at 42°C for 1 hour. β-actin was used as the internal control. The RT-PCR conditions were as follows: an initial denaturation at 95°C for 5 minutes, followed by 95°C for 25 seconds, 64°C for 20 seconds, and 72°C for 20 seconds to ensure the specificity; and 31 cycles at 93°C for 25 seconds, 60°C for 35 seconds, 72°C for 20 seconds were performed for data collection and sensitivity analysis, as detailed in previous study [17]. All of the partners which could be detected were attached as Supplementary Table 1.

### Efficacy evaluation

Tumors were evaluated during EGFR-TKIs treatment every 8 weeks, or were evaluated early when significant signs of progression appeared. Objective tumor responses were determined according to the Response Evaluation Criteria in Solid Tumors (RECIST 1.1). Objective responses rate (ORR) includes complete response (CR), partial response (PR), stable disease (SD) and progressive disease (PD). Disease control rate (DCR) is defined as the addition of objective response and stabilization rates (CR+ PR+SD).

### Statistical analysis

Survival curves were calculated using the Kaplan-Meier method from the start of diagnosis of advanced NSCLC until death or last follow-up. PFS of EGFR-TKIs was defined as the time from EGFR-TKIs therapy to documented progression or death from any cause. Statistical analysis was performed with the SPSS 16 software (Chicago, IL, US). *P* < 0.05 was considered statistically significant. The median follow-up period was 23.5 months (7.5-65) and the last follow-up was on April 31, 2015.

## SUPPLEMENTARY TABLE




