# Supplementary material for: Concurrent gene alterations with EGFR mutation and treatment efficacy of EGFR-TKIs in Chinese patients with non-small cell lung cancer
Source: Oncotarget. 2017 Feb 15;8(15):25046–54. doi: 10.18632/oncotarget.15337 (PMC5421908; doi:10.18632/oncotarget.15337)
Supplement: Supplementary file 1 [file oncotarget-08-25046-s001.pdf]

Concurrent gene alterations with EGFR mutation and treatment efficacy of EGFR-TKIs in Chinese patients with non-small cell lung cancer

SUPPLEMENTARY TABLE

Supplementary Table 1: Details of gene types in present study

See Supplementary File 1.
