# Supplementary material for: Concurrent gene alterations with EGFR mutation and treatment efficacy of EGFR-TKIs in Chinese patients with non-small cell lung cancer
Source: Oncotarget. 2017 Feb 15;8(15):25046–54. doi: 10.18632/oncotarget.15337 (PMC5421908; doi:10.18632/oncotarget.15337)
Supplement: Supplementary file 2 [file oncotarget-08-25046-s002.docx]

| Gene | Type |
| --- | --- |
| *ALK* | EML4 exon 13;ALK exon 20 |
|  | EML4 exon 6 ins 33;ALK exon 20 |
|  | EML4 exon 20;ALK exon 20 |
|  | EML4 exon 18;ALK exon 20 |
|  | EML4 exon 2;ALK exon 20 |
| *ROS1* | SLC34A2 exon4;ROS1 exon 32 |
|  | SLC34A2 exon14 del ;ROS1 exon 32 |
|  | CD74 exon6;ROS1 exon 32 |
|  | SDC4 exon2;ROS1 exon 32 |
|  | SDC4 exon4;ROS1 exon 32 |
|  | SLC34A2 exon4;ROS1 exon 34 |
|  | SLC34A2 exon14 del; ROS1 exon 34 |
|  | CD74 exon6; ROS1 exon 34 |
|  | SDC4 exon4; ROS1 exon 34 |
|  | EZR exon10; ROS1 exon 34 |
| *ROS1* | TPM3 exon 8;ROS1 exon 35 |
|  | LRIG3 exon 16;ROS1 exon 35 |
|  | GOPC exon 8;ROS1 exon 35 |
| *RET* | CCDC6 exon 1;RET exon 12 |
|  | NCOA4 exon9;RET exon 12 |
|  | KIF5B exon 15;RET exon 12 |
|  | KIF5B exon 16;RET exon 12 |
|  | KIF5B exon 23;RET exon 12 |
|  | KIF5B exon 22;RET exon 12 |
| G12D | KRASexon2 |
| G12S |  |
| G12A | KRASexon2 |
| G12V |  |
| G12R |  |
| G12C |  |
| G13C |  |
| A775_G776insYVMA | *HER2exon20* |
| A775_G776insYVMA |  |
| M774_A775insAYVM |  |
| G776>VC | HER2 exon20 |
| P780_Y781insGSP |  |
| G13R | NRAS exon 2 |
| G12C |  |
| G12V |  |
| G12A |  |
| G13V |  |
| Q61R | NRASexon3 |
| Q61K |  |
| Q61L |  |
| Q61H |  |
| H1047R | PIK3CA exon20 |
| E545K | PIK3CAexon9 |
| H1047L | *PIK3CAexon20* |
| E542K | PIK3CA exon9 |
| V600E | BRAF exon15 |
